# Thiol Peroxidases as Major Regulators of Intracellular Levels of Peroxynitrite in Live *Saccharomyces cerevisiae* Cells

**DOI:** 10.3390/antiox9050434

**Published:** 2020-05-16

**Authors:** André Luís Condeles, Fernando Gomes, Marcos Antonio de Oliveira, Luís Eduardo Soares Netto, José Carlos Toledo Junior

**Affiliations:** 1 Departamento de Química—Faculdade de Filosofia, Ciências e Letras de Ribeirão Preto, Universidade de São Paulo, Ribeirão Preto 14040-901, Brazil; acondeles@usp.br; 2 Departamento de Genética e Biologia Evolutiva, Instituto de Biociências, Universidade de São Paulo, São Paulo 05508-090, Brazil; fernandopgdna@hotmail.com (F.G.); nettoles@ib.usp.br (L.E.S.N.); 3 Instituto de Biociências, Campus do Litoral Paulista, Universidade Estadual Paulista Júlio de Mesquita Filho, São Vicente, São Paulo 11330-900, Brazil; mao@clp.unesp.br

**Keywords:** thiol peroxidases, peroxiredoxins, thioredoxins, peroxynitrite, boronate, nitric oxide, *Saccharomyces cerevisiae*

## Abstract

Thiol peroxidases (TP) are ubiquitous and abundant antioxidant proteins of the peroxiredoxin and glutathione peroxidase families that can catalytically and rapidly reduce biologically relevant peroxides, such as hydrogen peroxide and peroxynitrite. However, the TP catalytic cycle is complex, depending on multiple redox reactions and partners, and is subjected to branching and competition points that may limit their peroxide reductase activity in vivo. The goals of the present study were to demonstrate peroxynitrite reductase activity of TP members in live cells in real time and to evaluate its catalytic characteristics. To these ends, we developed a simple fluorescence assay using coumarin boronic acid (CBA), exploiting that fact that TP and CBA compete for peroxynitrite, with the expectation that higher TP peroxynitrite reductase activity will lower the CBA oxidation. TP peroxynitrite reductase activity was evaluated by comparing CBA oxidation in live wild type and genetically modified Δ8 (TP-deficient strain) and Δ8+TSA1 (Δ8 strain that expresses only one TP member, the *TSA1* gene) *Saccharomyces cerevisiae* strains. The results showed that CBA oxidation decreased with cell density and increased with increasing peroxynitrite availability. Additionally, the rate of CBA oxidation decreased in the order Δ8 > Δ8+TSA1 > WT strains both in control and glycerol-adapted (expressing higher TP levels) cells, showing that the CBA competition assay could reliably detect peroxynitrite in real time in live cells, comparing CBA oxidation in strains with reduced and increased TP expression. Finally, there were no signs of compromised TP peroxynitrite reductase activity during experimental runs, even at the highest peroxynitrite levels tested. Altogether, the results show that TP is a major component in the defense of yeast against peroxynitrite insults under basal and increasing stressful conditions.

## 1. Introduction

Peroxynitrite (ONOOH/ONOO^−^, pKa = 6.9) is an oxidant formed by a diffusion-controlled reaction of nitric oxide (NO^•^) with superoxide anion (O_2_^•−^) radicals (Equation (1), k_1_ = 1.9 × 10^10^ M^−1^ s^−1^) [1]. Peroxynitrite formation under normal conditions is minimized by keeping the NO^•^ and O_2_^•−^ concentrations low, but probably increases in episodes of infection and inflammation [2]. Upon protonation or reaction with CO_2_, peroxynitrite may generate other aggressive species, such as hidroxyl (OH^•^), nitrogen dioxide (NO_2_^•^), and carbonate anion (CO_3_^•−^) radicals (Equations (2) and (3)) [3,4]. Peroxynitrite and its downstream radical products potentially oxidize and damage many biological targets and may be involved in the onset of multiple pathophysiological conditions [5].

NO^•^ + O_2_^•−^ → ONOO^−^ (1)

ONOO^−^ + H^+^ → 0.3(NO_2_^•^ + OH^•^) + 0.7(NO_3_^−^ + H^+^) (2)

ONOO^−^ + CO_2_ → ONOOCO_2_^−^ → 0.35(NO_2_^•^ + CO_3_^•−^) + 0.65(NO_3_^−^ + CO_2_) (3)

Only a few cellular species have been identified as possible catalytic peroxynitrite scavengers, including members of the thiol peroxidase family [6]. Thiol peroxidases (TP), including peroxiredoxins (Prx) and glutathione peroxidases (GPx), are ubiquitous, abundant, and widespread proteins that react with a variety of biologically relevant peroxides and are, therefore, regarded as an important part of the cellular antioxidant repertoire [7]. TP may have different biological functions [7,8], but all of them essentially rely on their exceptional reactivity toward hydroperoxides.

**Scheme 1:**
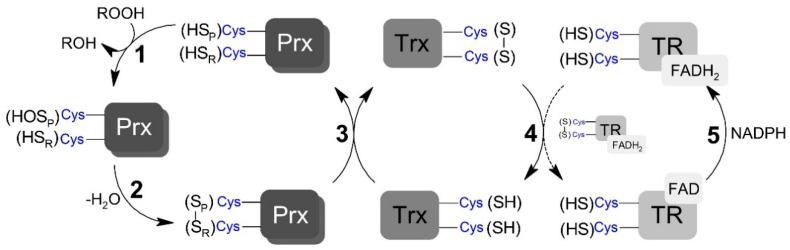
Generic representation of peroxiredoxins catalytic cycle. (1) Peroxidatic cysteine (S_P_H) oxidation to sulfenic acid (SOH) by peroxide and the formation of the leaving group (ROH). (2) Peroxiredoxin (Prx) disulfide (S_P_ – S_R_) formation by the condensation reaction between sulfenic acid and the resolving cysteine (S_R_H). (3) Prx disulfide reduction by thioredoxin (Trx) restoring the peroxidatic and resolving cysteine residues. (4) Reduction of oxidized Trx by thioredoxin reductase (TR). (5) Reduction of oxidized TR by NADPH (nicotinamide adenine dinucleotide phosphate; reduced form).

The assumption that TP detoxify peroxynitrite is based on the high rate constants by which TP members reduce peroxynitrite to the non-oxidant nitrite (NO_2_^−^) anion and in few studies addressing the growth or death of cells under exposure to peroxynitrite [9,10]. However, the catalytic cycle of TP is complex, depending on multiple redox reactions and partners (Scheme 1), a cascade of thiol-disulfide exchange reactions that may limit their peroxide reductase activities. Furthermore, most of evidence for cellular TP activity is from experiments exposing cells to oxidants and monitoring growth or are limited to laboratories that use molecular biology techniques, using genetically encoded probes involving a single TP (GFP-TP fusions) that focus on the monitoring of H_2_O_2_ [11]. As a consequence, there is a lack of temporal data describing how TP members globally handle hydroperoxides and peroxynitrite in real-time in live cells.

The goals of the present study were to demonstrate peroxidase activity of TP members in live cells in real time and to evaluate its catalytic properties. In order to address these needs, we employed a simple and readily accessible fluorescence spectroscopy-based competition assay using a peroxynitrite-reactive boronate compound to monitor peroxynitrite in real-time in *Saccharomyces cerevisiae* cells. The role of TP was investigated by comparing the boronate oxidation in live wild type and TP-deficient *Saccharomyces cerevisiae* cells with the expectation that higher TP peroxynitrite reductase activity will lower the coumarin boronic acid (CBA) oxidation.

## 2. Materials and Methods

### 2.1. Yeast Strains and Growth Conditions

The *Saccharomyces cerevisiae* strains employed for the present study include BY4741 (*MATa; his3∆1; leu2∆0; met15∆0; ura3∆0*); Tsa1/cTPxI YML028W (BY4741, *MATa, his3Δ1, leu2Δ0, met15Δ0, ura3Δ0, YML028w::kanMX4*), which were obtained from EUROSCARF (University of Frankfurt, Germany); as well as GY100 (*MATa, his3; leu2; met15; ura3;* *Δtsa1:KAN;* *Δtsa2:LEU2;* *Δdot5:MET15;* *Δahp1:HIS3;* *Δprx1:URA3;* *Δgpx2:HYG;* *Δgpx1:URA3;* *Δgpx3:KAN*), which were generous gifts from Vadim N. Gladyshev. The Δ8+TSA1 strain was constructed in a GY100 background in the pCEV-G1-Ph plasmid [12]. Strain construction was also described in Kaya et. al. [13].

*S. cerevisiae* strains were grown aerobically, at 30 °C in an incubator shaking at 150 rpm for 12 h, in YPD medium (1% yeast extract, 2% peptone, 2% dextrose). Under these conditions, the yeast were in the final mid-log phase at the time of the experiments. For the glycerol adaptation experiments, *S. cerevisiae* strains were grown as described above, centrifuged at 450 × *g* for 5 min, resuspended in YPG medium (1% yeast extract, 2% peptone, 3% glycerol), and incubated at 30 °C and 150 rpm for 4 h. After the second growth period, the yeast cells were harvested by centrifugation and resuspended in PBS buffer plus 0.01 mM diethylenetriaminepentaacetic acid (DTPA), pH 7.4, and kept in an ice bath at 5–8 °C. *S. cerevisiae* cells were viable before and after all experiments in the absence and in the presence (chemicals) on the basis of colony formation (not shown) and growth rate curves (Figure 8).

### 2.2. Chemicals 

Unless otherwise specified, all chemicals were purchased from Sigma-Aldrich and were of the highest purity available. Nitric oxide donor stock solutions, sper/NO (N-[4-[1-(3-aminopropyl)-2-hydroxy-2-nitrosohydrazino]butyl]-1,3-propanediamine) or deta/NO (2,2′-(hydroxynitrosohydrazono)bis-ethanimine), were prepared in 10mM NaOH and stored at −80 °C. The concentration of the donors was routinely measured using an oxyhemoglobin oxidation assay described elsewhere [14]. The stock solution of the fluorescent peroxynitrite indicator coumarin boronic acid (CBA; Cayman Chemicals) was prepared in DMSO and stored at −20 °C. The stock solutions of CBA and NO donors were prepared weekly and were diluted to minimize possible interference from the respective solvents. Paraquat (1,1′-dimethyl-4,4′-bipyridinium dichloride, PQ) solutions were freshly prepared in PBS, pH 7.4, before the experiments. The concentration of paraquat salt (PQ^2+^) stock solutions were spectrophotometrically determined using the strong absorption of the reduced radical form (PQ^+•^) at 600 nm (ε = 2.9 × 10^5^ L mol^−1^ cm^−1^) []. PQ^+•^ was prepared by reducing the salt form in a freshly prepared solution of 1% sodium dithionite NaOH 0.1 N [16].

### 2.3. Fluorescence Experiments

*S. cerevisiae* cells were harvested during the final log phase period, and the cell density was determined by measuring the absorbance of the cell suspension at 600 nm (OD_600_) in a UV-1800 spectrophotometer (Shimadzu). Prior to each experiment, aliquots from each *S. cerevisiae* culture were diluted to the required cell density using pre-warmed (30 °C) PBS buffer, pH 7.4, supplemented with 100 μM DTPA. Essentially, 20 × 10^6^ cells were loaded in each well of 96 micro-well plate, with a final volume of 250 µL). Next, CBA (10µM), paraquat (10µM), and the NO donor (sper/NO (50 µM) or deta/NO (500µM)) were introduced successively (see figure legends for more details). All of the experiments were performed at 30 °C using the microplate readers SpectraMax M3 or SpectraMax i3x (Molecular Devices) with excitation at 332 nm and emission at 456 nm. The slit width on both instruments was 9/15 nm for excitation and emission. Fluorescence acquisition was initiated immediately after the NO donor addition and recorded every 1 or 5 minute intervals after brief microplate shaking for at least 60 min. Additionally, between fluorescence recording cycles, the excitation slit was automatically closed to avoid 7-hydroxycoumarin (COH) photobleaching. 

### 2.4. SDS-PAGE and Western Blotting

Proteins were separated by SDS-PAGE. Routinely, proteins were transferred to nitrocellulose membranes (Amersham Biosciences Protran Premium, GE Healthcare) and incubated with primary antibodies (anti-porin (Thermo Fisher Scientific—16G9E6BC4), anti-Pgk1 (Nordic Immunology—NE130/7S), and anti-Tsa1 antibody (from F.G.’s lab). After washing the membranes, the specific secondary antibodies (anti-rabbit IgG, HRP-linked (Cell Signaling Technology) and anti-mouse IgG HRP-linked (Cell Signaling Technology)) were added. The immunoblots were developed using the ECL prime Western blotting detection reagent (GE Healthcare). The normalized band densitometry measurements were performed with the aid of the ImageJ software [17].

## 3. Results

### 3.1. The Competition Assay

**Scheme 2:**
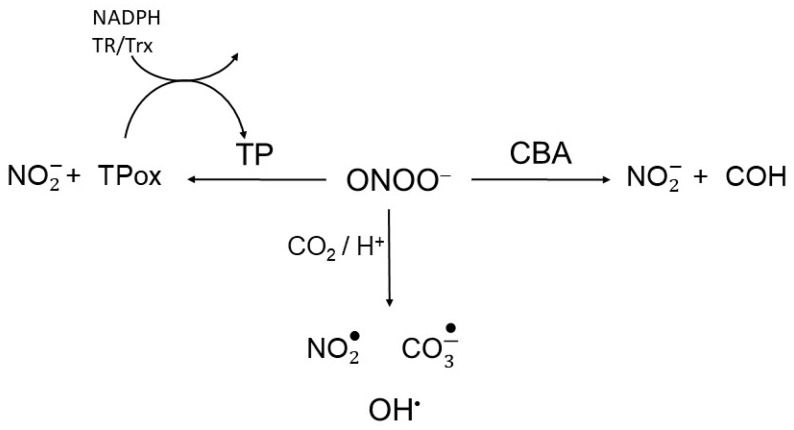
Peroxynitrite competition. Peroxynitrite is reduced to nitrite through reactions with thiol peroxidases (TP) and coumarin boronic acid (CBA), reacts directly with other target or undergoes CO_2_/H^+^-induced decomposition to radicals.

The basis for the peroxynitrite competition assay to test the intracellular TP peroxinitrite reductase activity in real-time is presented in Scheme 2. Briefly, cells were continuously exposed to a flux of peroxynitrite (ONOOH/ONOO^−^; pKa 6.9) in the presence of CBA, which rapidly and irreversibly (k = 1 x 10^6^ M^−1^s^−1^) reacts with peroxynitrite (Scheme 2, right arm). This reaction yields nitrite and the fluorescent product 7-hydroxy-coumarin (COH) [18]. Thus, in cells continuously exposed to peroxynitrite, TP and CBA will compete for peroxynitrite. Assuming that the levels of other cellular peroxynitrite reactive constituents remained unchanged, it was expected that the higher the TP peroxynitrite reductase activity would attenuate the rise of fluorescence by reducing the COH accumulation. The total peroxynitrite reductase activity of TP enzymes were accessed by comparing the CBA oxidation rates in live WT and TP-deficient *S. cerevisiae* strains [19]. 

*S. cerevisiae* was chosen as the model for the present study because this yeast expresses eight TP members (five Prx and three GPx) and viable strains lacking specific TPs, and all eight isoforms are available [19]. Additionally, *S. cerevisiae* also expresses a cytochrome *c* peroxidase (Ccp1), which has been shown to react rapidly with peroxynitrite [20].

Peroxynitrite fluxes were generated by combining an NO^•^ donor and paraquat (PQ), a redox cycler superoxide-generating compound [21]. This combination produces peroxynitrite through reaction between NO^•^ and O_2_^•−^ radicals [4], and is herein referred to as the PQ/NO^•^ donor. 

### 3.2. CBA Oxidation in Wild Type and Δ8 Yeast Cells during Peroxynitrite Generation

**Figure 1:**
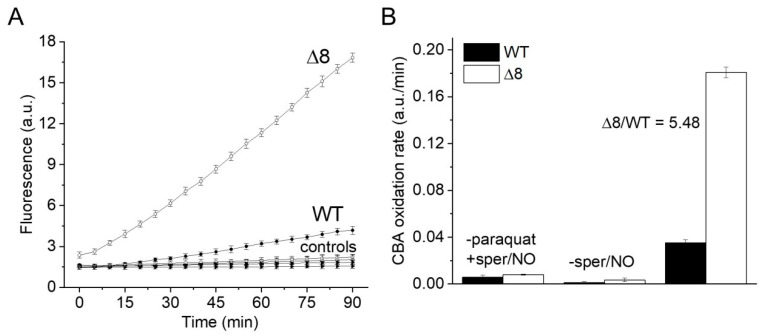
CBA oxidation in suspensions of WT and Δ8 *Saccharomyces cerevisiae* cells by peroxynitrite fluxes. WT and Δ8 *S. cerevisiae* cells were suspended in PBS supplemented with 100 μM DTPA (diethylenetriaminepentaacetic acid; pH 7,4) and 10 μM CBA, and placed in 96-well plates. Then, 10 μM paraquat and 50 μM sper/NO (PQ/NO^•^ donor) were added to selected wells, and the fluorescence measurements were initiated immediately and recorded every 5 min for 90 min. (**A**) Fluorescence of CBA oxidation with suspensions of WT and Δ8 yeast cells exposed to the PQ/NO^•^ donor. Controls refer to WT and Δ8 yeast samples lacking either PQ or NO^•^ donor. (**B**) The rate of peroxynitrite-mediated CBA oxidation generated by the PQ/NO^•^ in WT and Δ8 cells. The rate of CBA oxidation was defined as the slope of the fluorescence during the final 20 min of the experiment, at which time the fluorescence increase was linear. Each well contained 20 × 10^6^ cells. All experiments were performed in triplicate and repeated at least three times, yielding consistent results. Experiments were performed at 30 °C. Fluorescence was recorded under the following conditions: λ excitation = 332 nm and λ emission = 456 nm; slit width = 9/15 nm for both excitation and emission.

Suspensions of WT and Δ8 *S. cerevisiae* cells were transferred to wells of a 96-well plate and exposed to the PQ/NO^•^ donor and CBA. The fluorescence of COH was continuously monitored in a plate reader. There was a small CBA oxidation in control experiments in the presence of the NO^•^ donor (in the absence of PQ), probably due to cellular O_2_^•−^ production. Notably, CBA oxidation was negligible in control experiments in the absence of the NO^•^ donor (even in the presence of PQ), confirming that the changes in fluorescence were due to peroxynitrite under the experimental conditions of the study. Importantly, CBA oxidation rate increased significantly in cells exposed to PQ/NO^•^ donor and was always higher for the Δ8 strain (Figure 1A). These results are also shown as the rate of CBA oxidation (Figure 1B), which is defined in the Figure 1 legend.

PQ redox cycling depends on intracellular reducing agents [21] to generate O_2_^•−^, which is a short-lived species with limited diffusibility through biological membranes. Therefore, the observed peroxynitrite production associated with the PQ/NO^•^ donor must predominantly occur intracellularly, resulting in an environment where TP, other cellular components, and CBA compete for the oxidant. 

### 3.3. Effect of Cell Density and NO^•^ Donor Concentration on CBA Oxidation

**Figure 2:**
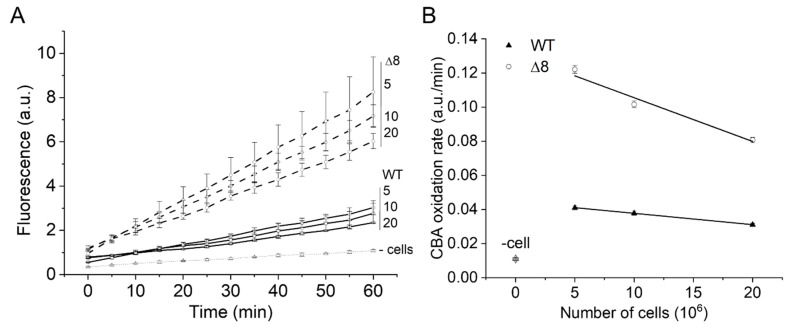
Effect of WT and Δ8 *S. cerevisiae* cell density on CBA oxidation. Fluorescence traces of CBA oxidation (**A**) and CBA oxidation rate (**B**) by peroxynitrite fluxes with increasing cell densities. CBA (10 μM), paraquat (10 μM), and deta/NO (2,2′-(hydroxynitrosohydrazono)bis-ethanimine; 500 μM) were added to 96-well plates containing a different number of WT and Δ8 *S. cerevisiae* cells per well (5, 10, and 20 × 10^6^). The fluorescence measurements were initiated immediately after the PQ/NO^•^ donor addition and were recorded every 5 min for 1 h. The experimental conditions and fluorescence acquisition parameters are described in the Figure 1 legend. The experimental procedures were performed at least three times with similar results.

The PQ/NO^•^ donor-induced CBA oxidation was negligible in cell-free experiments (Figure 2A, labeled as -cells), confirming that paraquat requires intracellular reducing agents for O_2_^•−^ production, and that O_2_^•−^ and peroxynitrite production occur primarily inside cells. In the presence of cells, the rate of CBA oxidation was higher in the Δ8 strain than the WT strain and decreased with increasing cell density for both WT and Δ8 strains (Figure 2A,B), indicating that peroxynitrite-dependent oxidation of CBA may have partially occurred in the extracellular space. 

Interestingly, the cell density-dependent effect on the rate of CBA oxidation was more pronounced for the Δ8 strain than the WT strain (Figure 2B), which is consistent with the peroxynitrite reductase activity of TP in WT competing better with extracellular CBA oxidation than that of Δ8. The pronounced effect of cell density for Δ8 strain also suggests the existence of a TP-independent peroxynitrite reductase component of the Δ8 strain (and of WT). 

**Figure 3:**
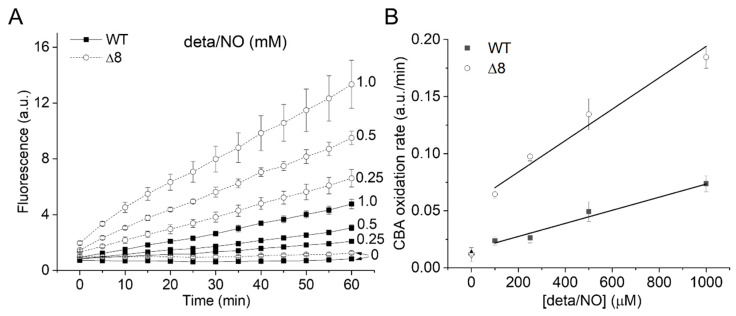
CBA oxidation in suspensions of WT and Δ8 *S. cerevisiae* cells as a function of NO^∙^ donor concentration. Fluorescence traces of CBA oxidation (**A**) and CBA oxidation rate (**B**) by peroxynitrite as a function of deta/NO concentration (0.1–1 mM). Suspensions of WT and Δ8 *S. cerevisiae* cells (20 × 10^6^ cells per well) were transferred to 96 well plates containing CBA (10 μM), and were exposed to paraquat (10 μM) and increasing concentrations of the deta/NO. Fluorescence measurements were recorded every five minutes for 60 min by a plate reader. The experimental conditions and fluorescence acquisition parameters are described in the Figure 1 legend.

The CBA oxidation rate increased linearly with NO^•^ donor concentration in both strains (Figure 3A), as expected from the competition between NO^•^ and SODs for O_2_^•−^, and was higher for the Δ8 strain at every NO^•^ donor concentration (Figure 3B). 

It is worth mentioning that the fluorescence increase was linear throughout the experiments using varying cellular densities and increasing NO^•^ donor concentrations, suggesting that the overall cellular peroxynitrite reductase activity of the WT and Δ8 strains remained unchanged throughout the experimental time scale. 

**Figure 4:**
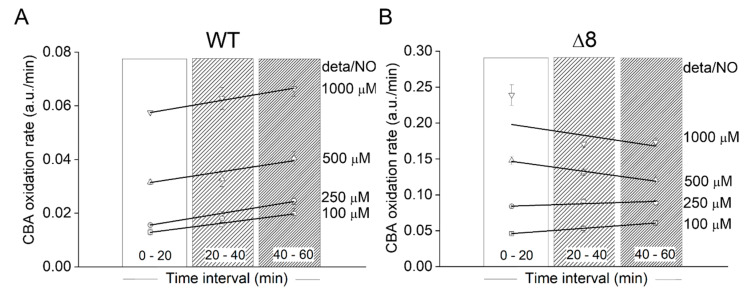
Rate of CBA oxidation in suspensions of WT and Δ8 *S. cerevisiae* cells as a function of the time and NO^•^ donor concentration. (**A**) WT *S. cerevisiae.* (**B**) Δ8 *S. cerevisiae*. Suspensions of WT and Δ8 *S. cerevisiae* cells in 96-well plates (20 × 10^6^ cells per well) containing CBA (10 µM) were exposed to paraquat (10 μM) and increasing concentrations of deta/NO. The rate of CBA oxidation was determined by the linear regression of the data within each time interval shown. The experimental conditions and fluorescence acquisition parameters are described in the Figure 1 legend. The data are from a representative experiment carried out in triplicate and repeated three times with similar results.

Using the data of Figure 3A, the CBA oxidation rate was also plotted as a function of deta/NO concentration during three different time periods for WT (Figure 4A) and Δ8 (Figure 4B) strains. There was only a slight acceleration in the CBA oxidation rate over time for the WT strain at all NO^•^ donor concentrations tested, again suggesting that the peroxynitrite reductase activity of WT *S. cerevisiae* cells remained virtually constant throughout the experimental run and that TP catalytically remove peroxynitrite. TP were also probably the limiting reactant. Overall, under the experimental conditions, the bulky (considering the intra and extracellular space) TP concentration was roughly between 0.04 and 0.5 µM (this calculation was performed assuming 100 µM TP, an intracellular concentration purposely exaggerated, and a *S. cerevisiae* diameter between 2 and 5 µm). Using a standard fluorescence analytical curve, the accumulated COH concentration after 1 h for the highest NO^•^ donor concentration employed was estimated to be around 0.8 and 2.4 µM for the WT (Figure 3A) and Δ8 (Figure 3B) strains, respectively. It should be pointed out that this estimation did not take into account the peroxynitrite consumed by cellular targets other than CBA (including TP) since the CBA concentration used was not saturating. Thus, it was likely that TP catalytically reduced peroxynitrite under the experimental conditions, as total peroxynitrite production during experimental runs was greater than TP concentrations. 

A similar behavior was observed at the two lowest deta/NO concentrations with the Δ8 strain. For the two highest donor concentrations, for reasons that are not clear, negative slopes were observed. It could have been due to CBA exhaustion or fluorescent detector saturation.

It was also observed that the CBA oxidation rate nonlinearly increased with PQ concentration (data not shown). This result was probably due to O_2_^•−^ production by PQ depending on other intracellular factors, such as cellular PQ take up, and O_2_ and/or PQ-reducing cellular component availabilities.

### 3.4. Oxidation of CBA in S. cerevisiae Adapted to Glycerol Medium

**Figure 5:**
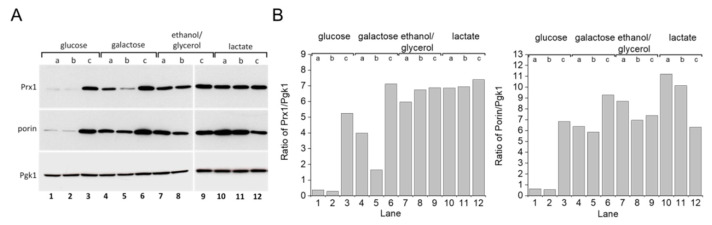
Mitochondrial protein expression in *S. cerevisiae* grown with different carbon sources and growth phases. (**A**) Immunoblotting of porin and Prx1 in yeast WT cells grown in different carbon sources. (**B**) Comparative densitometry of porin and Prx1 from panel (A). Yeast WT cells were grown in different carbon sources and collected in three different growth phases, as determined by the OD_600_. Cell extracts were prepared and proteins were separated by SDS-PAGE gel electrophoresis followed by immunoblotting with specific antibodies at the (a) early exponential (OD_600_ =v1.0), (b) mid–late exponential (OD_600_ = 2.0), and (c) stationary (OD_600_ 2.0) growth phases. Pgk1 protein was used as the gel loading control. All experiments were performed in triplicate and repeated at least three times, yielding consistent results.

**Figure 6:**
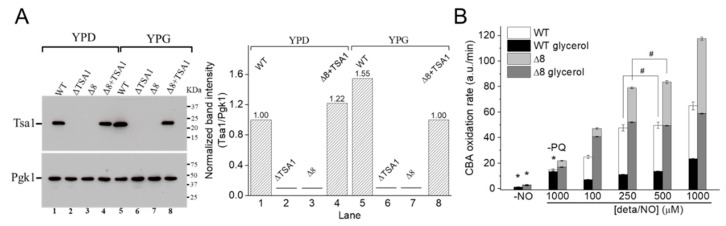
CBA oxidation in suspensions of glycerol-adapted WT and Δ8 *S. cerevisiae* cells. (**A**) Immunoblotting and comparative densitometry of the Tsa1 in selected strains grown in YPD and YPG mediums. (**B**) The rate of CBA oxidation of control and glycerol-adapted WT and Δ8 *S. cerevisiae* cells. CBA (10 μM), paraquat (10 μM), and increasing concentrations of deta/NO were added to 96-well plates containing 20 × 10^6^ WT or Δ8 *S. cerevisiae* cells per well. Experimental conditions and fluorescence acquisition parameters are described in the Figure 1 legend. The experimental procedures were performed at least three times with similar results.

*S. cerevisiae* grown in glucose-rich medium [22,23,24] rely mostly upon fermentation to produce ATP. When glycerol is substituted for glucose as a carbon source, the yeast depend more on mitochondrial respiratory metabolism. One response yeast employ for adapting to glycerol-rich medium involves mitochondrial biogenesis [25]. Accordingly, we detected increased levels of the outer mitochondrial membrane protein porin (Figure 5A, lanes 4–12; Figure 5B, bottom panel), which has been used as a mitochondrial biogenesis marker [26]. A similar result was observed with cells grown in galactose or lactate-rich media. Increased porin expression was also observed in the stationary growth phase of cells grown in glucose (Figure 5A, lane 3; Figure 5B, bottom). Previous studies have demonstrated that growing *S. cerevisiae* cells in these other carbon sources generates more mitochondrial oxidants and that the yeast, in turn, adapt by modulating the expression levels of antioxidant enzymes such as superoxide dismutase, catalase, and TP [27,28,29]. Indeed, mitochondrial peroxiredoxin 1 (Prx1) expression levels increased in yeast cells grown in media containing galactose, ethanol/glycerol, or lactate when compared to cells grown in glucose (Figure 5A,B, top panel). Additionally, the WT strain displayed increased TSA1 protein expression levels when grown in glycerol (Figure 6A,B, compare lanes 1 and 5).

It was hypothesized that once the WT strain was glycerol-adapted, the peroxynitrite availability would be lower than that in WT cells grown in glucose, both because of increased prevention of peroxynitrite formation (by induction of SODs expression) and increased TP expression. To this end, experiments such as those described in Figure 2 and Figure 3 were conducted with WT and Δ8 glycerol-adapted cells. 

Control experiments in the absence of PQ (Figure 6B, labeled –PQ) detected a slight decrease in the rate of CBA oxidation in glycerol-adapted cells when compared to the respective control group. As expected, in the presence of PQ, the CBA oxidation rate increased with NO^•^ donor concentration in both control and glycerol-adapted cells. Additionally, glycerol-adapted WT cells had reduced CBA oxidation rates relative to control cells over the entire range of NO^•^ donor concentrations tested (Figure 6B), which is indicative of reduced peroxynitrite availability. Interestingly, the CBA oxidation rate of the glycerol-adapted Δ8 strain was also lower than the respective control cells (Figure 6B). This finding is possibly related to decreased peroxynitrite formation and may also indicate that a TP-independent peroxynitrite reductase activity was present in these cells and that this system is up-regulated under respiratory conditions.

### 3.5. Oxidation of CBA in Δ8+TSA1 S. cerevisiae Strain

**Figure 7:**
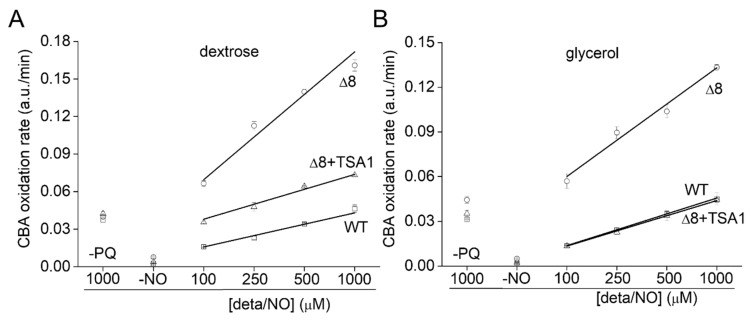
Rate of CBA oxidation in suspensions of Δ8+TSA1 *S. cerevisiae* cells. (**A**) Rate of CBA oxidation by peroxynitrite fluxes in different *S. cerevisiae* strains. (**B**) Rate of CBA oxidation by peroxynitrite fluxes in different glycerol adapted *S. cerevisiae* strains. The labels -PQ and -NO respectively refer to a set of experiments excluding paraquat and the NO^•^ donor as controls. The experimental conditions and fluorescence acquisition parameters are described in the Figure 1 legend.

To further evaluate the role of TP as peroxynitrite reductase, we performed experiments with the Δ8+TSA1 strain. This yeast strain expresses the *TSA1* gene under the control of a constitutive promoter (the endogenous translation elongation factor, TEF-1 alpha) [30]. The expression of TSA1 by the Δ8+TSA1 strain was confirmed at the protein level, and was comparable to the TSA1 expression levels in the WT strain (Figure 6A, compare lanes 1 and 4). Notably, Δ8+TSA1 was significantly more resistant to peroxynitrite-dependent CBA oxidation when compared to the Δ8 strain (Figure 7A), and the CBA oxidation rate of the glycerol-adapted Δ8+TSA1 cells was indistinguishable from the glycerol-adapted WT strain (Figure 7B). These observations further show that TP is an important peroxynitrite reductase system. 

### 3.6. The Growth of Different S. cerevisiae Strains Challenged with Peroxynitrite Fluxes

**Figure 8:**
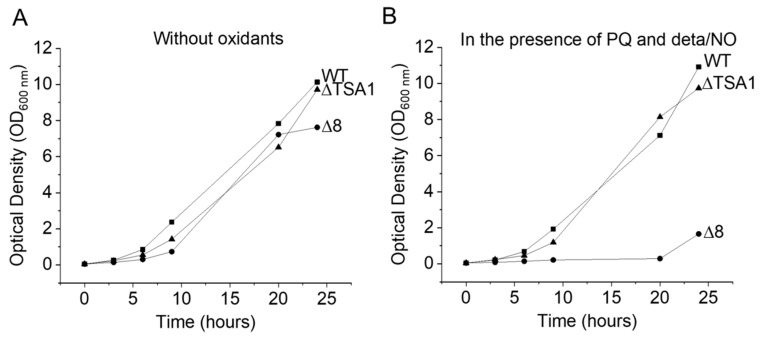
Growth curves of different *S. cerevisiae* strains under normal and stress conditions. (**A**) Growth of WT, Δ8, and ΔTSA1 strains in YPD. (**B**) Growth of WT, Δ8, and ΔTSA1 strains in YPD in the presence of PQ/NO^•^ donor.

The growth of *S. cerevisiae* strains exposed to peroxynitrite fluxes generated by the PQ/NO^•^ donor were monitored for 24 h (Figure 8A,B). Under simulated normal conditions, the growth of the WT, ΔTSA1, and Δ8 strains was similar. However, when exposed to peroxynitrite fluxes, the Δ8 strain was drastically affected. These results are in agreement with earlier data [9,31] showing that TP members are important peroxynitrite scavengers. 

## 4. Discussion

The TP catalytic cycle is complex and subjected to numerous side reactions. TP members reduce peroxynitrite (and other different hydroperoxides) with high rate constants via a conserved peroxidatic cysteine (C_P_), which is oxidized to sulfenic acid in the process (Scheme 1, step 1). Depending on the TP member and redox conditions, this sulfenic acid species can have different fates. For example, the sulfenic acid can be overoxidized by the hydroperoxide. However, a condensation reaction between the resulting Cp sulfenic acid and a second conserved residue called the resolving cysteine (C_R_), yielding a disulfide bridged form (Scheme 1, step 2), typically occurs following oxidation [32]. The disulfide TP forms can also have different fates, activating signaling events through covalent interactions with other proteins, including transcription factors [33], or be fully reduced back to the resting peroxide reactive form via thiol-based oxidoreductase partners (Scheme 1, step 3) [34,35,36]. In *Saccharomyces cerevisiae*, the oxidized form of both Prx and GPx are reduced, through thiol-disulfide exchange reactions, by each one of the three oxidoreductases called thioredoxin (Trx) [37]. The resulting disulfide formed in Trx is subsequently reduced via NADPH-dependent thiol–disulfide exchange reactions with the flavoenzyme thioredoxin reductase (TR) (Scheme 1, steps 4 and 5; reviewed in [38]). TP-mediated reduction of peroxynitrite is rapid, with rate constants varying between 10^6^ and 10^8^ M^−1^ s^−1^ [10,39,40], depending on TP member and substrate. Few previous studies measuring the rate constant of step 2 (Scheme 1) have shown that this reaction significantly varies among TP members [37,38,41,42], but it is typically a rapid unimolecular first-order process, which is complete within a few seconds. The kinetics of the thiol–disulfide exchange reaction downstream from the resolution step has only been assessed for a limited number of redox partners, with rate constants ranging from 10^5^ to 10^6^ M^−1^ s^−1^ [40,43,44,45]. 

Although the several steps of the TP catalytic cycle exhibit high reaction rate constants, TP peroxide reductase turnover is directly dependent on the kinetics of each successive step, the concentrations of downstream redox partners, and on the aforementioned branching points of the whole catalytic cycle species. Perturbations in any of these parameters could potentially limit TP peroxide reductase catalytic activity. For example, steady-state NADPH oxidation measurements in the presence of the entire TP system (Scheme 1) underestimated the rate constant of the reaction between the Cp of several Prx enzymes and different peroxides (step 1) by 3 to 4 orders of magnitude, possibly because experiments were performed with limited concentrations of the downstream redox partners. Only more direct kinetic approaches revealed the exceptionally high rate constants for this step. In cells, the TP catalytic system may be limited still by other parameters such as the substrate and/or TP compartmentalization, expression of TP redox system members, redox partner competition between TPs and other cellular oxidases, cellular redox status, and Cp hyperoxidation [32,33]. Furthermore, it has been demonstrated that TP enzymes undergo structural switches, from a fully-folded decamer to a locally unfolded dimer, during catalysis [32,46]. Individually or collectively, these variables represent numerous potential bottlenecks for the TP peroxide reductase activity in living cells. Accordingly, Bayer et al. [47] have suggested that Prx 2 remove hydrogen peroxide from red blood cells in a stoichiometric manner. 

In the present study, we developed a fluorescence-based competition assay that is capable of monitoring, in real-time, the TP-mediated peroxidase activity in live cells. The assay can be used to measure TP peroxynitrite reductase activity in presumably any cell type, provided that the appropriate control experiments are performed. The CBA approach also allowed us to make some other interesting observations. The ability of our CBA assay to monitor peroxynitrite in real time gave us evidence that TP members constitute a relevant and catalytic peroxynitrite reductase system in living *S. cerevisiae* cells. Accordingly, the CBA oxidation rate (i.e., fluorescence increase) was linear throughout most of the 60–90 min time span of the experiments, with no signs of partway through acceleration of CBA oxidation, suggesting that TP levels and activities were not compromised during experimental runs, even though the accumulated peroxynitrite formed exceeded total TP concentration. These results are in line with TP kinetic properties and add the fact that the potential cellular bottlenecks factors do not limit the peroxynitrite activity to the point where removal of the peroxides becomes stoichiometric in *S. cerevisiae* cells under different respiratory metabolic and stressful conditions.

Similar results would likely be observed with other biologically relevant TP substrates, such as hydrogen peroxide. In addition, the conclusion that TP catalytically scavenge peroxynitrite and other peroxides may be cautiously extrapolated to mammalian cells, whose TP reactivity, kinetic properties, and catalytic cycle are not fundamentally different from yeast TP. The CBA competition assay could in theory be used to test this expectation using simple cellular models. Such peroxynitrite reductase activity of mammalian cells is particularly important for activated macrophages, potentially helping sustain their functions during episodes of infection and inflammation, where peroxynitrite formation is likely and a long-lasting, macrophage-mediated immune response is critical. 

The Δ8 strain results suggest the existence of a TP-independent peroxynitrite reductase species. It was previously shown, using a fundamentally different experimental approach, that Δ8 strain survival and growth under oxidative conditions is strictly dependent on mitochondrial Ccp1 heme-protein [30]. The ferric species of this protein rapidly reduces hydrogen peroxide and peroxynitrite (to nitrite) and is concomitantly oxidized to a compound 1-like heme-peroxidase, which is fully reduced back to the ferric, peroxynitrite reactive species by two successive one-electron reductions by mitochondrial ferrous cytochrome c [36], and is thus highly dependent on the reducing power of the mitochondria. Interestingly, the Δ8 strain that naturally acquired an extra copy of chromosome XI, where the CCP1 gene is located (consequently increasing Ccp1 protein expression), was positively selected. As well as this, genetic deletion of chromosome XI or the CCP1 gene, specifically, is lethal to the Δ8 strain [30]. Thus, Ccp1 apparently could serve as a backup system when the TP catalytic cycle is compromised [30]. In the present study, we showed that CBA oxidation in Δ8 strain exposed to fluxes of peroxynitrite decreases with increasing cell densities (Figure 2) and attenuates in glycerol adapted Δ8 cells when compared to Δ8 control cells (Figure 6B). Taken together, these results suggest that a TP-independent peroxynitrite reductase system exists in Δ8 strain (in WT strain), and that it is upregulated under respiratory conditions, which is consistent with a role for Ccp1. However, our data indicate that Ccp1 cannot fully compensate for TP deficiency, as the levels of CBA oxidation were always noticeably higher for the Δ8 when compared to the WT strain. 

### 5. Conclusions

The CBA competition assay can be used to study the specific or global peroxynitrite reductase activity of TP in real time in live cells comparing CBA oxidation in genetically modified cells. The assay provided a global picture of the peroxynitrite metabolism in living *S. cerevisiae*, showing that TP sustain a catalytic peroxynitrite reductase activity under simulated normal and increasingly stressful conditions.
